# Recognizing Rare Sequelae of Epstein-Barr Virus Myocarditis Leading to Dilated Cardiomyopathy and Acute Congestive Heart Failure With Multivalvular Regurgitation

**DOI:** 10.7759/cureus.21504

**Published:** 2022-01-22

**Authors:** Mina Aknouk, Suhrim Choe, Harmony Osborn, Anish Kanukuntla, Priyaranjan Kata, Arthur Okere, Pramil Cheriyath

**Affiliations:** 1 Internal Medicine, Hackensack Meridian Health Ocean University Medical Center, Brick, USA; 2 Cardiology, Hackensack Meridian Health Ocean University Medical Center, Brick, USA

**Keywords:** epstein-barr virus, myocarditis, dilated cardiomyopathy, lv systolic dysfunction, valvular regurgitation

## Abstract

Myocarditis is associated with a wide range of infections, most commonly viral (cytomegalovirus), bacterial, and parasitic (*Trypanosoma cruzi*). Epstein-Barr virus (EBV) rarely causes myocarditis, which is a life-threatening complication. Autoantibodies against cardiac myocytes activate the complement system and cause diffuse myocyte necrosis. Myocarditis has a variable presentation from asymptomatic to cardiogenic shock. Over time, untreated myocarditis can progress and result in dilated ventricles. Continued dilation of ventricles leads to systolic dysfunction, conduction abnormalities, ventricular arrhythmia, heart failure, valvular abnormalities, and thromboembolism. So, we are emphasizing the importance of early diagnosis and treatment of EBV to prevent mortality. This case study represents a rare case of mortality secondary to EBV infection with resultant DCM and congestive heart failure (CHF).

## Introduction

Myocarditis is primarily a diagnosis of exclusion, with variable presentations that may be similar to multiple cardiologic etiologies. Confirmation of viral myocarditis is difficult, with cardiac biopsy being the gold standard for diagnosis. With continued inflammation of the myocardium, myocarditis results in the remodeling of the ventricular structure, thinning and weakening the myocardium leading to dilated cardiomyopathy (DCM) [1]. DCM is characterized by reduced ejection fraction and dilation of the left ventricle (LV). While viral causes are the most common causes of myocarditis, a variety of infectious and inflammatory sources are also at fault including toxic ingestion, drug hypersensitivity, autoimmune disease, and bacterial, fungal, or parasitic infections [1]. Amongst the viral causes in Europe and the United States (US), coxsackievirus and parvovirus are the most common. Epstein-Barr virus (EBV) is one of the more rare causes. Parvovirus B19 is suspected to be responsible for 51.4% of myocarditis with DCM in a German study while EBV was associated with 2% of cases [2]. 

EBV, also known as Human Herpes Virus-4 (HHV4), belongs to the *Herpesviridae* family. It infects 90% of the global population, with primary infection during childhood and remaining latent into adulthood, often reemerging during immunocompromised times [3]. Initial infection is common during childhood with mild viral symptoms or asymptomatic infection. In adolescents and adults, it often presents with symptoms of infectious mononucleosis (IM). EBV remains latent in B lymphocytes and memory cells. From here it can lead to a wide range of cancers, including gastric and nasopharyngeal carcinomas, B cell neoplasms such as Hodgkin's lymphoma (HL), diffuse large B cell lymphoma (DLBCL), and Burkitt lymphoma [4]. Myocarditis and DCM are rare but life-threatening consequences of EBV. This study analyzes a case of EBV-related myocarditis in a 20-year-old immunocompetent male, who developed severe left ventricular systolic dysfunction with reduced ejection fraction (dilated cardiomyopathy) resulting in mortality.

## Case presentation

**Figure 1:**
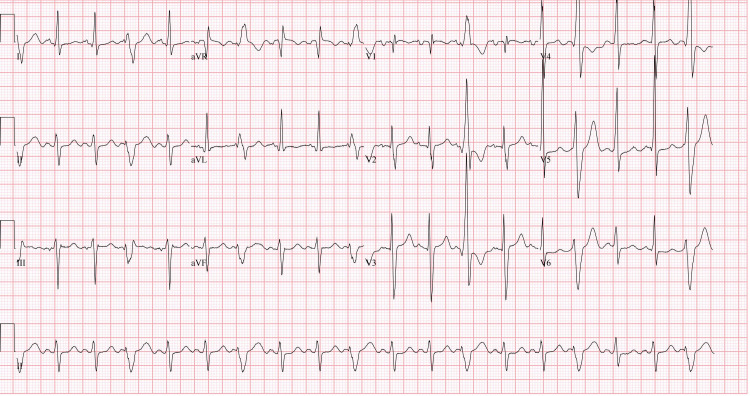
ECG Changes

A 20-year-old male was admitted to the hospital for right upper quadrant abdominal pain, jaundice, nausea, vomiting, and mild shortness of breath (SOB). Pain radiates to the chest with no prior history of similar symptoms. He had no sick contact or recent travel history. Vital signs showed no fever with a temperature of 98.8 degrees Fahrenheit, hypotension with a blood pressure of 78/54 mmHg, tachycardia with a pulse of 126 beats per minute, tachypnea with a respiratory rate of 33 respirations per minute, and pulse oximetry of 95% on room air. Cardiac examination revealed tachycardia, with a normal S1, S2, and S3 present without murmur. Pulmonary examination revealed coarse breath sounds, bibasilar wheezing, and bilateral diffuse crackles throughout bilateral lung fields on lung auscultation. The patient’s abdomen was distended with mild diffuse tenderness on palpation. Lower extremities showed 1+ bilateral pitting edema. Chest x-ray findings showed moderate cardiomegaly and small pericardial effusion with diffuse mosaic attenuation throughout the lung parenchyma. ECG showed sinus tachycardia with frequent premature ventricular complexes and fusion complexes, possible left atrial enlargement, left axis deviation with nonspecific ST and T wave changes with left ventricular hypertrophy with QRS widening (Figure 1).

**Figure 2:**
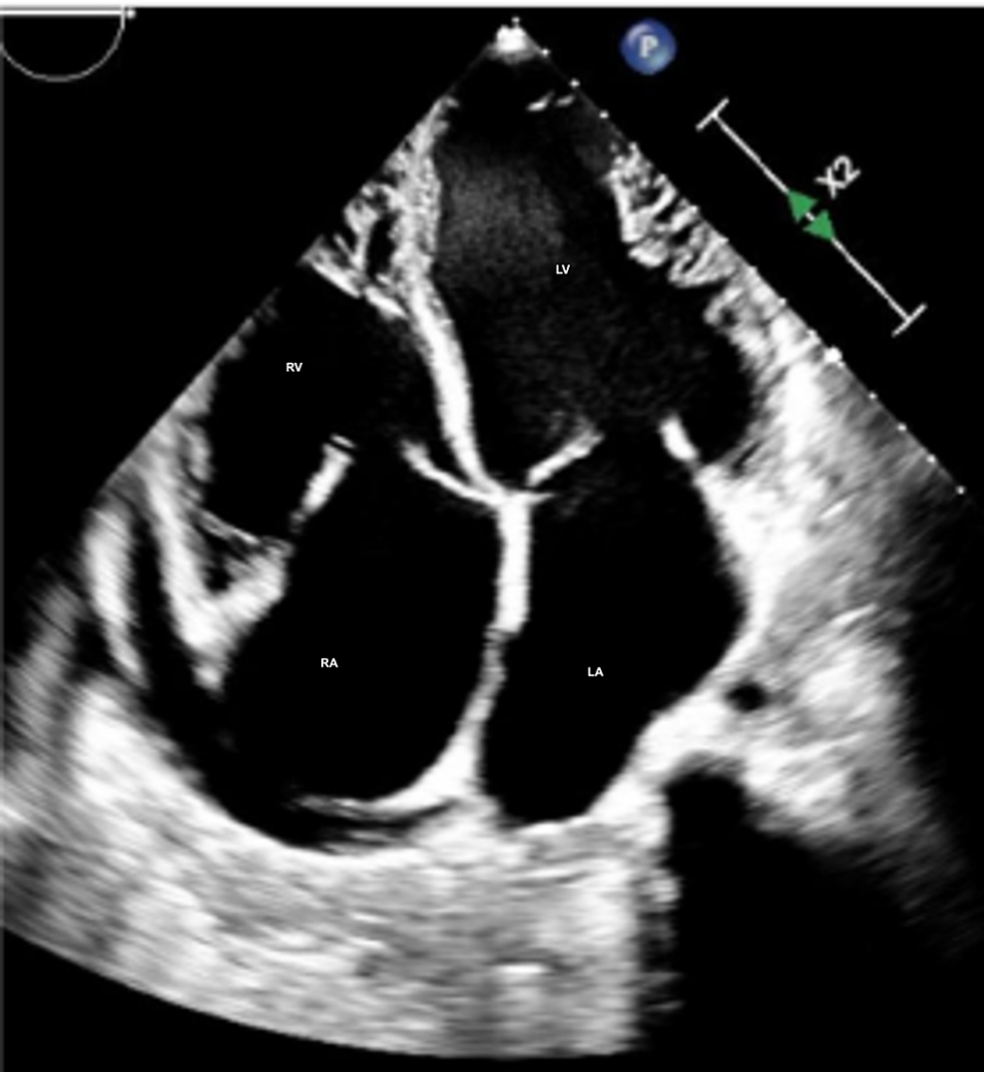
Left Ventricle (four-chamber view on echocardiogram) LV: left ventricle; RV: right ventricle; LA: left atrium; RA: right atrium

**Figure 3:**
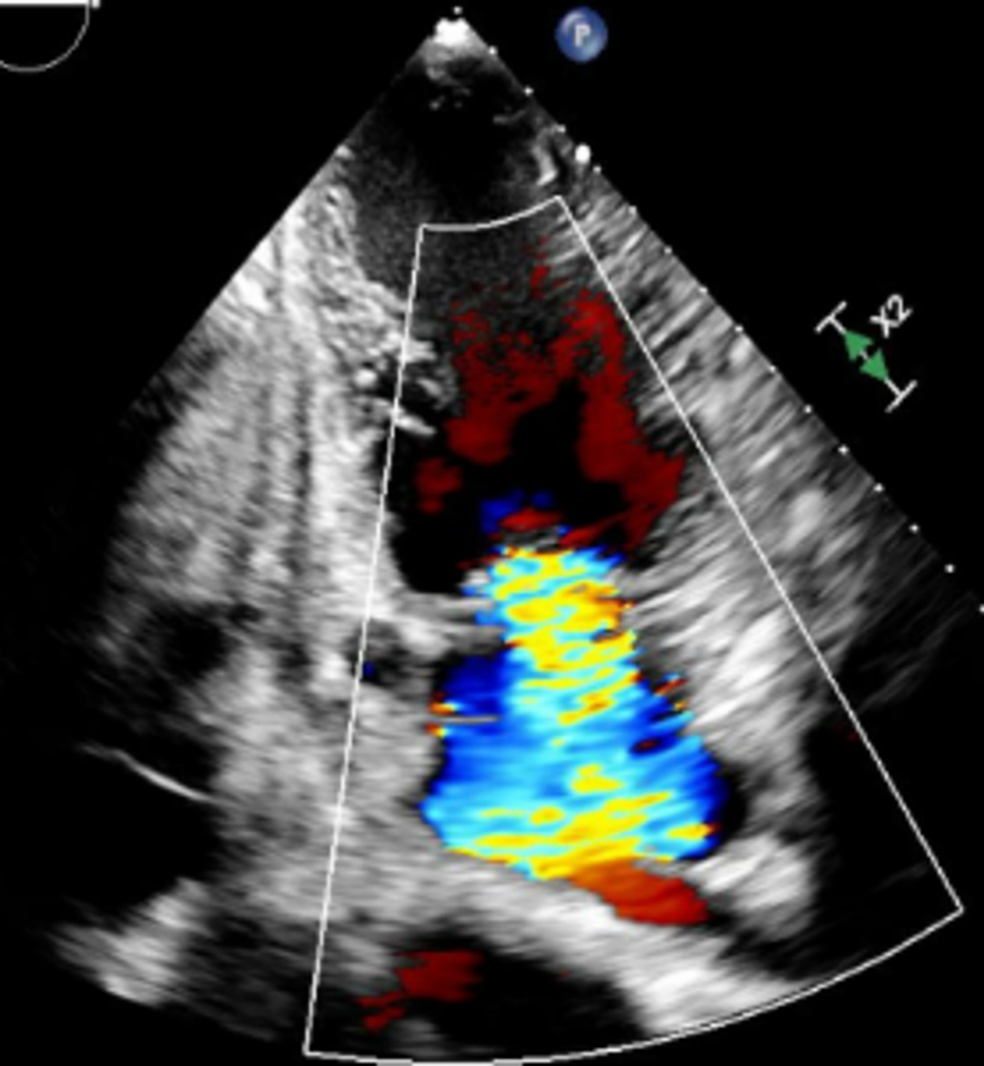
Mitral Regurgitation (two-chamber view on echocardiogram)

**Figure 4:**
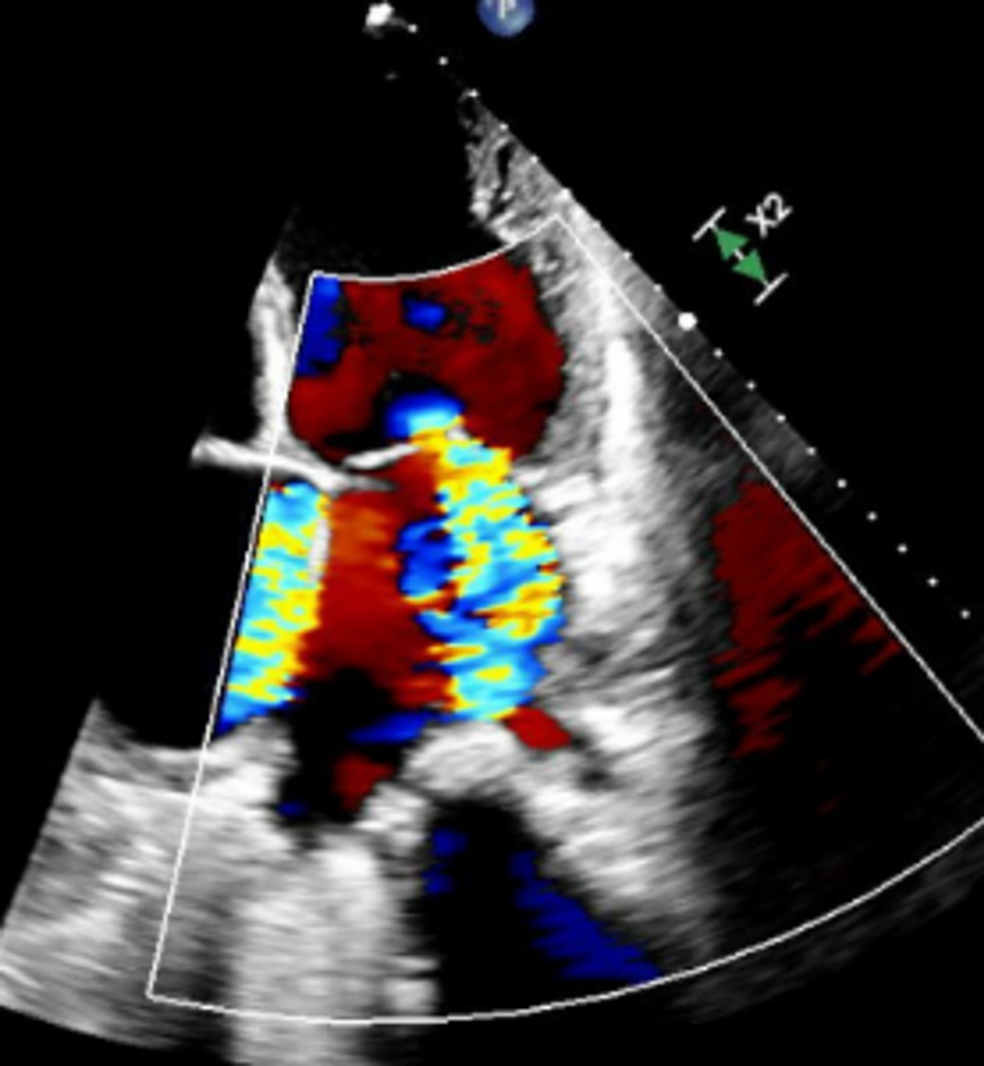
Mitral Regurgitation (four-chamber view on echocardiogram)

**Figure 5:**
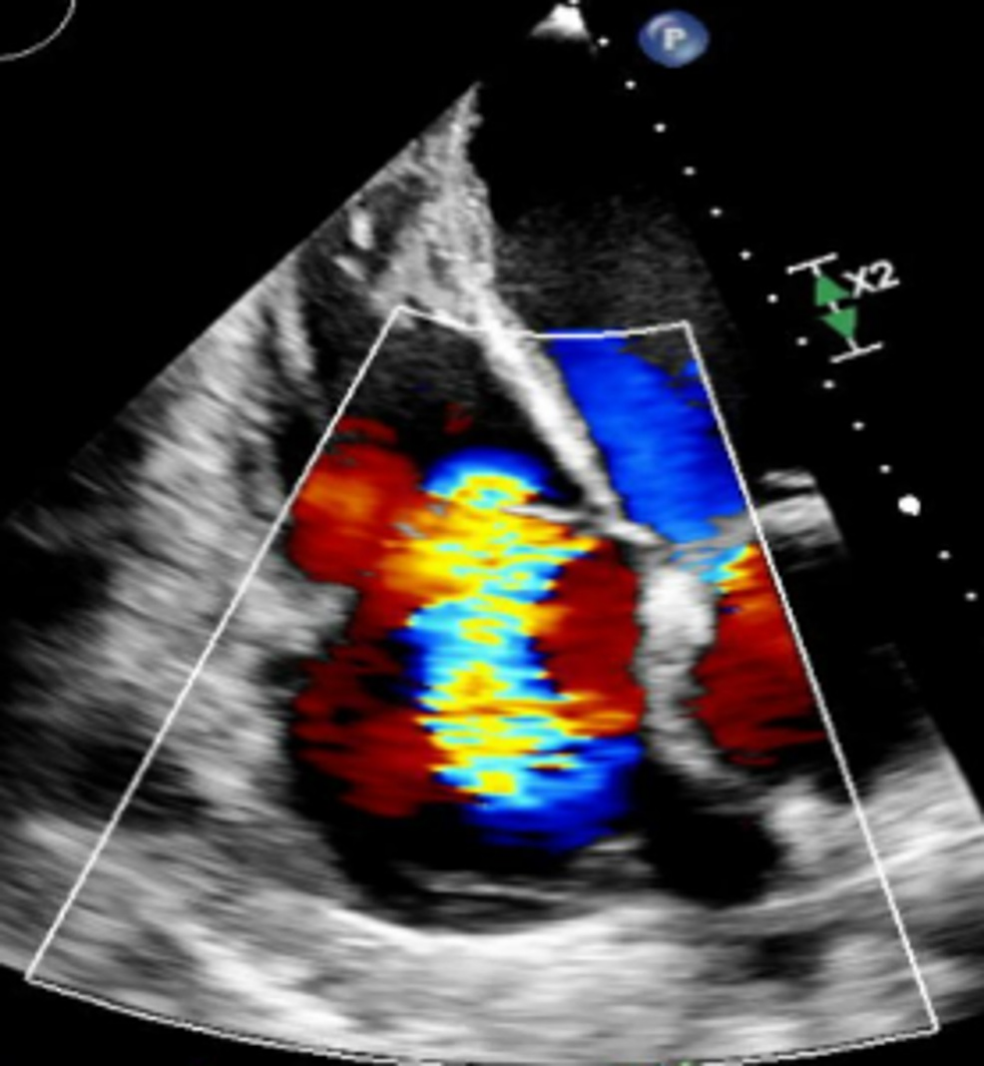
Tricuspid Regurgitation (four-chamber view on echocardiogram)

Hematologic analysis was notable for anemia (hemoglobin of 9.3 g/dL (13.2-17.5 g/dL)). The comprehensive metabolic panel, including liver function test (LFT) and renal function, was within normal limits. B-type natriuretic peptide (BNP) was markedly elevated at 1749 pg/ml (<100 pg/ml). An echocardiogram (Video 1, Figures 2-5) demonstrated an ejection fraction of 12% with acute severe systolic heart failure and moderate to severe mitral regurgitation, tricuspid regurgitation, and pulmonary hypertension. Echocardiography showed severely increased left ventricular cavity, severe global hypokinesis. 

**Video 1:** Echocardiogram

The patient was admitted to the intensive care unit (ICU) and treated with dobutamine, furosemide, spironolactone, midodrine, and tolvaptan. During the patient's time in the ICU, he underwent multiple thoracenteses for extensive bilateral pleural effusions to alleviate respiratory distress and hypoxia. Multiple abdominal paracenteses were performed for ascites, which he had developed as a result of his acute liver failure secondary to liver congestion. A viral panel was completed to assess for possible infectious causes and returned a positive result for EBV. Cardiac catheterization and implantable cardiac defibrillator placement were considered but the patient was not stable enough for any invasive measures. Considering this patient’s age and good health prior to this admission, he was deemed to be a good candidate for cardiac transplant. However, the patient was not a US citizen and had minimal social support available, which prevented him from being added to the transplant list. Multiple tertiary centers were contacted to alleviate this social situation. The Guatemalan embassy was also contacted; however, the patient refused to return to Guatemala for a cardiac transplant. The patient’s clinical status deteriorated rapidly, and the decision was made to intubate the patient but he developed cardiogenic shock. Development of multiple organ failure followed soon after. Near the end of his life, the patient suffered from hepatic encephalopathy, severe ascites, and coagulopathy. Albumin deficiency led to extravasation of fluid to the interstitial space and worsening hypotension. The patient became anuric and fluid overloaded from severe renal failure secondary to the cardio-renal syndrome. All efforts made to correct the electrolyte imbalance were unsuccessful. The patient eventually developed cardiac arrhythmia incompatible with life and was unresponsive to resuscitation, resulting in death. 

## Discussion

EBV is a double-stranded (ds) DNA virus and belongs to the *Herpesviridae* family. EBV is the primary cause of IM, a common infection worldwide with a lifetime prevalence of 90% [4]. EBV can result in various complications such as hepatitis, splenic rupture, airway compromise, lymphoproliferative disorders, multiple sclerosis, rheumatoid arthritis, and chronic active EBV. Myocarditis is a rare complication of acute and chronic EBV infection.

Myocarditis is generally caused by direct viral invasion as well as autoimmune and toxic mechanisms. Autoantibodies against the myocytes activate the complement system and then cause cellular cytotoxicity [5]. This causes diffuse myocyte necrosis and the remaining myocardium dilates to compensate [6]. Progressive dilatation of the ventricles results in tricuspid and mitral valve insufficiency. Compensatory tachycardia and increased peripheral vascular resistance further reduce the ejection fraction and increase the ventricular wall stress and end-systolic volumes, resulting in worsening symptoms [7]. 

Patients infected with EBV can present initially with prodromal viral illness features such as fever, coryza, and rhinorrhea. Patients who have progressed to DCM have a long latent period where they will remain asymptomatic until ejection fraction becomes reduced and symptoms become evident [7]. Patients commonly present with symptoms of heart failure including orthopnea, paroxysmal nocturnal dyspnea (PND), peripheral edema, cough, progressive exertional dyspnea, palpitations, and impaired exercise tolerance. Transient complete heart block occurs occasionally in a few patients and the implantation of a permanent pacemaker is needed in rare cases to correct arrhythmia [8]. Our patient presented with atypical features of heart failure including jaundice, nausea, vomiting, and right upper quadrant pain.

In acute myocarditis, erythrocyte sedimentation rate (ESR) and C-reactive protein (CRP) are typically elevated. Additionally, elevated white blood cell (WBC) count and creatine kinase are non-specific findings suggestive of myocarditis. Elevated titers of antibodies against cardiotropic viruses are also found. The chest x-ray may show an enlarged cardiac silhouette with pulmonary venous congestion [7]. Electrocardiography most commonly shows sinus tachycardia, diffuse ST-T wave changes, prolonged QT interval, ventricular tachyarrhythmia, and conduction abnormalities in acute myocarditis. Left ventricular dimensions can be normal in the acute infection phase as evidenced by echocardiography. However, in some patients, a trabeculated pattern of the interventricular septum and ventricular walls can be found in the early stages of myocarditis when the inflammation is substantial [7]. As myocarditis progresses, diffuse hypokinesis and left ventricular dilation develops due to systolic dysfunction with reduced ejection fraction <30% [9]. Cardiovascular magnetic resonance imaging is a non-invasive and valuable test in the diagnosis of myocarditis. The T2-weighted edema imaging is used to assess the presence of acute myocardial inflammation. Late gadolinium enhancement (LGE) imaging reveals either an intramural, rim-like pattern in the septal wall or a subepicardial (patchy) distribution of myocardial damage in the free left ventricular lateral wall. However, LGE imaging cannot differentiate between acute and chronic inflammation, showing damaged myocardium without specificity [10].

One study shows that there is a significant elevation of EBV antibody titers in patients with ischemic heart disease when compared to the healthy subjects [11]. However, a definitive diagnosis can be made only by an invasive endomyocardial biopsy and identifying viral genome by PCR techniques. Myocardial biopsy is indicated once echocardiography and angiography confirm no other likely etiology of cardiomyopathy with a clinically decompensating patient. Two-dimensional echocardiography findings revealing a dilated ventricle (left or right), global hypokinesis, reduced left ventricular ejection fraction of less than 40% with unidentified etiology can support the decision of biopsy. Coronary angiography must show absent ischemic pathology with no severe disease as well [6].

Hematologic evaluation of our patient revealed anemia and an elevated brain natriuretic peptide (BNP) level. Echocardiography showed severe left ventricle (LV) systolic dysfunction with an ejection fraction of 12% and moderate to severe mitral and tricuspid regurgitation. During his one-month stay in the ICU, a viral panel was positive for EBV.

Treatment of EBV with interferon (IFN)-beta and IFN-alpha2 protects myocytes from injury and reduces inflammatory cell infiltrates. IFN-beta treatment resulted in almost complete elimination of cardiac viral load in some cases [10]. The treatment of dilated cardiomyopathy due to EBV involves the use of intravenous diuretics to relieve volume overload. Angiotensinogen converting enzyme (ACE) inhibitors, angiotensin receptor blockers (ARB), beta-blockers such as carvedilol, and long-acting metoprolol have been found useful in patients with reduced ejection fraction (EF) [7]. Cardiac glycosides such as digoxin, calcium channel blockers, and aldosterone antagonists can also be used to treat heart failure. In this patient, sacubitril/valsartan (Entresto) use was contraindicated because of the patient’s fulminant acute renal failure.

Anticoagulants such as low molecular weight heparin and warfarin are used in patients with atrial fibrillation or valvular heart disease occurring concomitantly with viral myocarditis induced DCM. Cardiac transplantation is the definitive treatment when all the other therapies fail. An implantable cardiac defibrillator (ICD) is placed to prevent sudden cardiac death (SCD) in patients who are poor candidates for transplant [10]. Our patient did not respond to heart failure therapy and remained unstable for any invasive intervention for the majority of his admission. The patient did not recover and developed cardiac arrhythmia incompatible with life and died despite aggressive medical management.

## Conclusions

Although cardiomyopathy and myocarditis are rare complications of EBV, it is important to consider them in the setting of a young adult with acute decompensated CHF. Biopsy and cardiac magnetic resonance imaging are essential in confirming the diagnosis of myocarditis. In the presence of heart failure, progression is likely and cardiac transplantation is one of the few definitive treatments. Recovery is possible with early identification and management, highlighting the importance of the recognition of this diagnosis.
